# Ownership, Use of, and Interest in Digital Mental Health Technologies Among Clinicians and Young People Across a Spectrum of Clinical Care Needs: Cross-sectional Survey

**DOI:** 10.2196/30716

**Published:** 2022-05-11

**Authors:** Imogen H Bell, Andrew Thompson, Lee Valentine, Sophie Adams, Mario Alvarez-Jimenez, Jennifer Nicholas

**Affiliations:** 1 Orygen Parkville Australia; 2 Centre for Youth Mental Health University of Melbourne Parkville Australia; 3 Division of Mental Health and Wellbeing Warwick Medical School University of Warwick Coventry United Kingdom; 4 Austin Health Melbourne Australia

**Keywords:** adolescent, youth mental health, mental health, attitude, digital technology, internet-based interventions, digital mental health, mental health services, clinician, mobile phone

## Abstract

**Background:**

There is currently an increased interest in and acceptance of technology-enabled mental health care. To adequately harness this opportunity, it is critical that the design and development of digital mental health technologies be informed by the needs and preferences of end users. Despite young people and clinicians being the predominant users of such technologies, few studies have examined their perspectives on different digital mental health technologies.

**Objective:**

This study aims to understand the technologies that young people have access to and use in their everyday lives and what applications of these technologies they are interested in to support their mental health. The study also explores the technologies that youth mental health clinicians currently use within their practice and what applications of these technologies they are interested in to support their clients’ mental health.

**Methods:**

Youth mental health service users (aged 12-25 years) from both primary and specialist services, young people from the general population (aged 16-25 years), and youth mental health clinicians completed a web-based survey exploring technology ownership, use of, and interest levels in using different digital interventions to support their mental health or that of their clients.

**Results:**

A total of 588 young people and 73 youth mental health clinicians completed the survey. Smartphone ownership or private access among young people within mental health services and the general population was universal (611/617, 99%), with high levels of access to computers and social media. Youth technology use was frequent, with 63.3% (387/611) using smartphones several times an hour. Clinicians reported using smartphones (61/76, 80%) and video chat (69/76, 91%) commonly in clinical practice and found them to be helpful. Approximately 50% (296/609) of the young people used mental health apps, which was significantly less than the clinicians (*χ^2^*_3_=28.8, n=670; *P*<.001). Similarly, clinicians were significantly more interested in using technology for mental health support than young people (H_3_=55.90; *P*<.001), with 100% (73/73) of clinicians being at least slightly interested in technology to support mental health compared with 88% (520/591) of young people. Follow-up tests revealed no difference in interest between young people from the general population, primary mental health services, and specialist mental health services (all *P*>.23). Young people were most interested in web-based self-help, mobile self-help, and blended therapy.

**Conclusions:**

Technology access is pervasive among young people within and outside of youth mental health services; clinicians are already using technology to support clinical care, and there is widespread interest in digital mental health technologies among these groups of end users. These findings provide important insights into the perspectives of young people and clinicians regarding the value of digital mental health interventions in supporting youth mental health.

## Introduction

Digital mental health interventions (DMHIs) are interventions that use technologies, such as smartphones, smartwatches, or computer programs, to provide information, support, or treatment for mental health, most commonly using the internet [1]. As either standalone self-help tools or those used in conjunction with standard care in the form of *blended therapy* [2], DMHIs have the potential to support mental health and well-being [3]. Interest in DMHIs often centers on young people because of the high prevalence of mental health difficulties in this age group coupled with their frequent use of technology [4-6]. Indeed, the potential to access mental health support via technology may be particularly important for young people, given that their access and sustained engagement with mental health services are limited [7,8].

The role of technology in supporting mental health was made starkly clear by the global COVID-19 pandemic. During this time, many nations became reliant on technology-enabled service delivery to provide mental health care at a distance [9,10]. For many, telehealth has become the norm, enabling direct client contact via telephone or videoconferencing [11]. This unique moment in history has catalyzed an important shift in the perceived value of technology-supported care. However, although research indicates that telehealth can be as effective as face-to-face treatment and may improve service quality in the eyes of young people with mental health difficulties [12,13], it only represents the tip of the iceberg of how technology can support mental health. In the wake of the pandemic, there exists an opportunity to capitalize on the increased interest and acceptance of technology-enabled care to deliver new digital interventions that not only provide a more convenient way of delivering treatment but also the potential to enhance it [14].

Despite the potential of DMHIs, a lack of long-term engagement has often been reported. This is true both in clinical trials [15] and, particularly, in naturalistic studies in which apps are used *in the wild* [16], where good initial uptake is commonly followed by a dramatic drop in use over time [15]. Poor engagement with DMHIs has been highlighted as a significant problem in the field and has formed the focus of several reviews [17-20]. A common theme identified in this literature is a lack of fit between evidence-based DMHIs and the needs of end users for whom they are designed to help [20]. A lack of emphasis on understanding end user needs has resulted in an early generation of DMHIs that have generally lacked relevance and interest for users. Critical learnings from this early work have resulted in the greater emphasis placed on involving end users in the design process, as well as a need for research dedicated to understanding their needs and preferences. Young people constitute a particular type of end user who tends to be highly exposed to technologies in daily life, making them particularly critical of digital products [19]. Therefore, understanding the unique perspectives of young people is important to inform the development of DMHIs for youth.

A shift toward practices that prioritize the needs and preferences of young people as end users is required to ensure that DMHIs are engaging and fit for purpose [19,21]. However, there is currently a dearth of research on the technologies that young people are interested in using to support their mental health. Qualitative studies have explored experiences with DMHIs among young people, finding preferences toward their use to support, rather than replace, face-to-face services, as well as a desire to tailor digital interventions to individual preferences [22-24]. Quantitative findings in youth populations are limited, although 2 studies in small samples of young people in early psychosis services found high levels of technology ownership and use in these populations [25,26] and an interest in technology for a variety of purposes to support self-management and functional recovery [25]. Although these findings provide some insight into technology use and preferences, the qualitative findings are limited in generalizability, and quantitative research has involved young people with specific mental health conditions. To fill this gap, this study aims to understand what technologies young people, both within youth mental health services and in the general population, have access to and use in their everyday lives, and which applications of these for supporting their mental health they are most interested in. Furthermore, as DMHIs are most effective when combined with human support [1,27,28], a highly likely use case is the blending of these tools within youth mental health services. Despite this, there are very few examples of the successful implementation of DMHIs within clinical settings, highlighting the significant gap between research and practice in digital mental health [29]. As such, in addition to young people, this study aims to investigate the use of and interest in different DMHIs among clinicians in youth mental health services to support their clinical work.

## Methods

### Ethics Approval

The study was approved by the Melbourne University human research ethics committee (approval numbers 2057299 and 2056793) and the Melbourne Health human research ethics committee (reference number QA2020096) and complied with the Declaration of Helsinki.

### Study Design and Context

Young people and mental health clinicians completed a web-based survey as part of the BRACE project, which examined the effects of COVID-19 on the mental health and well-being of young people living in Australia, telehealth service quality, and the potential of technology to support youth mental health care. Data collection for the project occurred during and immediately after Australian Federal and State government–mandated lockdown restrictions (*stage 3*) that included *socially distancing* from individuals not part of a household and limited ability to leave home [30]. During the lockdown and the following months, most mental health services shifted to telehealth delivery [9,13].

This study reports primary findings on access to technologies and the use of and interest in different technologies for mental health support among young people and clinicians. Young people, aged between 12 and 25 years, were recruited through 2 sources. As part of a larger survey on social media and self-harm, the survey was advertised to the general population of young people aged 16 to 25 years on social media between June and October 2020. Young people who had scheduled an appointment between March 23, 2020, and August 7, 2020, at Australian primary (headspace) or specialist youth mental health services in Victoria or Queensland were also sent an SMS text message invitation to complete the survey. In Australia, headspace is the leading primary youth mental health service funded by the Australian Federal Government via the Primary Health Networks to provide early intervention for young people aged 12 to 25 years with mild to moderate and high-prevalence mental health conditions [31]. A smaller number of specialist services offer care to young people aged 12 to 25 years with more complex, low-prevalence disorders. Notably, care for early presentations of psychosis is provided by specialist tertiary-level mental health services. The specialist service is unique in Australia in size and extends across one-third of the Melbourne metropolitan area. Finally, clinicians who provided youth mental health care at these same services during the same period also received a link to complete a version of the survey.

### Procedure

All participants completed the web-based survey using Qualtrics XM (Qualtrics). In the general population, after clicking the survey link, interested potential participants were screened for eligibility (aged between 16 and 25 years and residing in Australia). The survey was conducted on June 11, 2020, and was open for approximately 4 months. Eligible young people (aged 12-25 years) from 4 primary headspace services in Victoria were identified via the appointment calendars of the participating services. On May 28, 2020, an anonymous web-based survey link was sent via SMS text message to all those with appointments, and a reminder SMS text message was sent 2 weeks later. Young people from specialist services in Victoria and Queensland were provided the link by SMS text message, email, or letter between May 28 and June 11 (Victoria) and July 28 and August 7 (Queensland). Using a clinical staff email list, clinicians were sent a link to the anonymous web-based survey on May 10, 2020 (Victoria), and July 13, 2020 (Queensland), and given approximately 2 weeks to complete it.

### Measures

#### Overview

In consultation with young people, the surveys were created specifically for the BRACE project, with young people and clinician surveys covering identical themes. Measures related to this study aimed to understand (1) access to and use of technology for mental health and (2) levels of interest in technologies to support mental health care among young people and clinicians.

#### Technology Access and Use

Technology access and use were explored by asking young people if they owned or had private access to various technologies, ranging from smartphones and laptops to social media and gaming consoles. Those who indicated that they had access to the technology were asked how often they used it on a Likert scale ranging from *less than once a week* to *several times an hour*. Similarly, clinicians were asked if they had used the same technologies in their clinical practice. For the technologies they had used, they rated how helpful they thought the technology was for their clients. Both young people and clinicians were asked whether they had used a mental health app or recommended a smartphone app for their clients’ mental health. Young people who had used apps to support their mental health were asked which apps they had used and to rate their helpfulness. Clinicians who had recommended apps to their clients were asked to name the apps they had recommended and rate how helpful they were for their clients.

#### Technology Interest

The level of interest in using 20 different technologies commonly used to support mental health was measured on a 5-point Likert scale ranging from *not at all interested* to *extremely interested*. Technologies ranged from established resources such as telehealth, websites, and helplines to emerging digital mental health tools such as virtual reality (VR), serious games, and chatbots. Young people rated their interest in using each technology to support their mental health, whereas clinicians rated their interest in using or recommending each technology to support the mental health and well-being of their clients.

All quantitative items were measured on Likert scales, with anchors varying depending on the question, as specified in the results. A full copy of the survey is provided in Multimedia Appendix 1.

#### Mental Health Measures

The Patient Health Questionnaire-4 [32] was used to characterize the mental health status of the participants in the sample. The Patient Health Questionnaire-2 (PHQ-2) is a 2-item, brief self‐report screening questionnaire for clinical depression. Similarly, the Generalized Anxiety Disorder-2 (GAD-2) is a 2-item brief self-report screening questionnaire for clinical anxiety. Items are on both measures rated on a 4-point Likert type scale from 1 (not at all) to 4 (nearly every day). The total scores range from 0 to 6, with higher scores indicating greater levels of depression or anxiety. A score of ≥3 on the 2-item PHQ-2 indicates probable depressive disorder, and a score of ≥3 on the 2-item GAD-2 indicates probable anxiety disorder for adults and young people in primary care settings and the general population [32,33].

### Data Analysis

Quantitative data were analyzed using descriptive statistics in SPSS (version 22.0, IBM). Owing to the focus of the paper, the survey was considered complete if participants responded to the technology interest items; however, all available data were reported, and pairwise analyses were performed. Owing to this, and as survey items were not mandatory, the sample size varied between analyses and is reported where it differed. Chi-square statistics were used to examine differences among participant groups (young people from the general population, young people from primary mental health services, young people from specialist mental health services, and clinicians) and the use of apps for mental health. To gain an indication of participants’ overall interest in technology to support mental health, overall interest in technology was calculated as the mean of an individual’s interest scores across the 20 technology types. Kruskal-Wallis tests with Bonferroni-corrected follow-up contrasts were used to examine differences among participant groups in terms of overall interest in using technology to support mental health. Similar technologies were grouped to examine differences among participant groups concerning interest in technology types. The following seven groups were formed by the research team based on the original 20 technology items:

1. Web-based self-help (web-based therapy, mental health websites, and web-based employment support)
2. Mobile self-help (apps to support mental health, apps to track mental health, and wearables to track mental health such as smartwatches)
3. Telehealth (video chat with clinician, telephone with clinician, texting with clinician, and mental health support lines)
4. Blended therapy (blended therapy and sharing mental health information with clinicians on the web)
5. Social media (secure social media to connect with young people about mental health and social media to connect with clinicians about mental health)
6. Immersive technologies (VR for mental health strategies, augmented reality for mental health strategies, VR with clinicians, and virtual worlds for mental health groups)
7. Interactive technologies (chatbots for mental health support and digital games for mental health support)

## Results

### Sample Characteristics

Within primary care services, an SMS text message link to the survey was sent to 1868 young people, 308 (16.49%) of whom responded to the survey, and of the 308 respondents, 229 (74.4%) completed it. Within specialist services, the survey was distributed to approximately 650 young people, of whom 59 (9.1%) responded, and of these 59 respondents, 53 (90%) completed it. The survey was also advertised on social media, and of the 693 people who clicked the link, 498 (71.9%) provided consent and were eligible, and of those who were eligible, 306 (61.4%) completed the survey items reported in this study. Finally, of the approximately 370 clinicians who received the survey link, 92 (25%) initiated the survey, and of those 92 clinicians, 73 (79%) completed it. The final sample comprised 73 clinicians across specialist and primary services and 588 young people (age range 12-25 years) from primary care, specialist services, and the general population. Demographic characteristics of the youth sample are shown in Table 1.

**Table 1:** Characteristics of young people from the general population, primary services, and specialist services (N=588).

Characteristics			General population (n=306)		Primary services (n=229)		Specialist services (n=53)
Age (years), mean (SD)			21.20 (2.90)		18.77 (3.48)		21.08 (2.54)
**Gender, n (%)**							
	Female	222 (72.5)		142 (62.0)		26 (49)	
	Male	58 (19)		63 (27.5)		26 (49)	
	Transgender	1 (0.3)		10 (4.4)		0 (0)	
	Nonbinary	14 (4.6)		7 (3.1)		1 (2)	
	Unspecified	11 (3.6)		7 (3.1)		0 (0)	
Aboriginal or Torres Strait Islander			6 (2)		4 (1.7)		1 (2)
**Current living situation, n (%)**							
	Living with parents, caregivers, or siblings	201 (65.7)		191 (83.4)		39 (74)	
	Living with friends	29 (9.5)		3 (1.3)		0 (0)	
	Living with romantic partner	30 (9.8)		11 (4.8)		0 (0)	
	Living in shared accommodation	23 (7.5)		9 (3.9)		5 (9)	
	Living alone	23 (7.5)		14 (6.1)		4 (8)	
	Homeless or couch surfing	0 (0)		1 (0.4)		3 (6)	
**State of residence, n (%)**							
	ACT^a^	11 (2.4)		0 (0)		0 (0)	
	New South Wales	31 (10.1)		0 (0)		0 (0)	
	Northern Territory	1 (0.2)		0 (0)		0 (0)	
	Queensland	16 (5.2)		0 (0)		16 (30)	
	South Australia	10 (3.3)		0 (0)		0 (0)	
	Tasmania	17 (5.6)		0 (0)		0 (0)	
	Victoria	211 (69.0)		229 (100)		37 (70)	
	Western Australia	9 (2.9)		0 (0)		0 (0)	
**Employment status,^b^ n (%)**							
	Full-time student	182 (59.5)		126 (55.0)		13 (25)	
	Part-time student	35 (11.4)		15 (6.6)		3 (6)	
Hours of study each week, mean (SD)			24.98 (12.22)		22.14 (17.96)		16.43 (8.77)
Full-time paid employment, n (%)			54 (17.6)		13 (5.7)		3 (6)
Part-time paid employment, n (%)			103 (33.7)		34 (14.8)		9 (17)
Hours of work each week, mean (SD)			23.35 (13.33)		19.72 (12.75)		24.02 (11.68)
Unpaid worker as a parent or carer, n (%)			6 (2.0)		1 (0.4)		1 (2)
Currently unemployed, n (%)			43 (14.1)		72 (31.4)		30 (57)
**Mental health,^c,d^ n (%)**							
	Potential clinical depression	133 (43.5)		69 (62.7)		30 (57)	
	Potential clinical anxiety	152 (49.7)		65 (59.1)		31 (60)	

^a^ACT: Australian Capital Territory.

^b^Categories are not mutually exclusive.

^c^Patient Health Questionnaire-2 and Generalized Anxiety Disorder-2.

^d^A score of ≥3 on the 2-item depression and anxiety screening measures indicates probable depressive or anxiety disorder (n=110).

### Technology Access and Use

#### Access and Use of Technology by Young People

**Figure 1:**
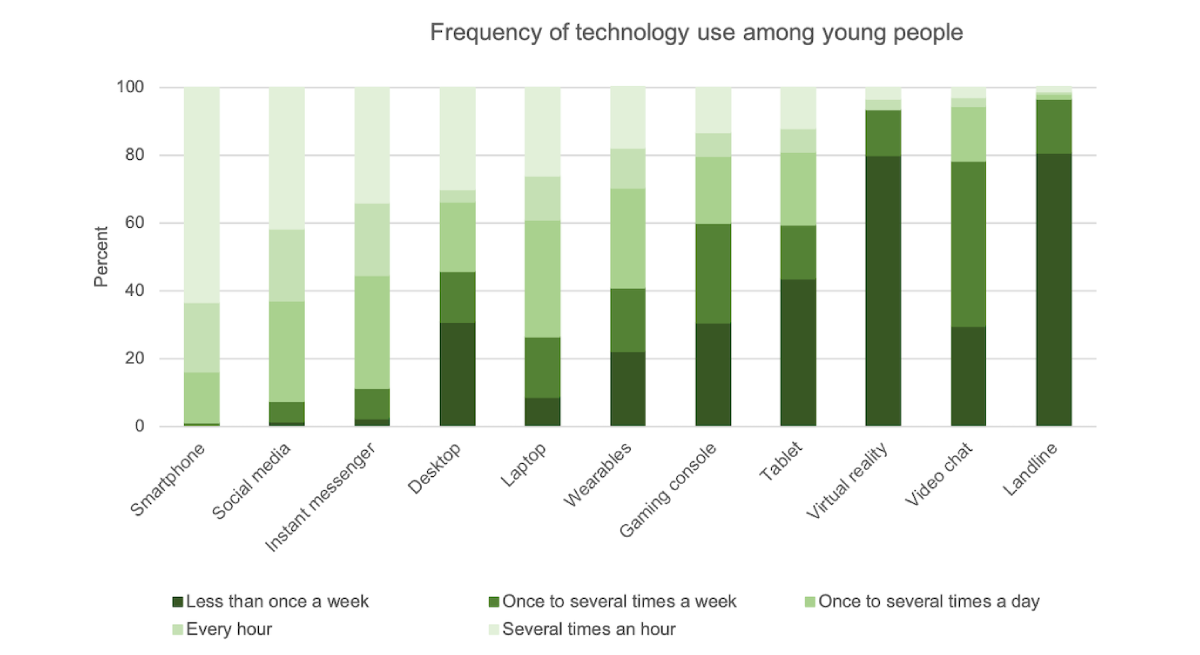
Young people’s average frequency of use across technologies that they have access to (as presented in Table 2).

Young people’s access to different technologies is displayed in Table 2. Smartphone access was universal (611/617, 99%), including among young people from primary and specialist services. Across the groups, young people reported high rates of video chat, instant messenger, and social media access and lower levels of access to wearable technologies and VR. Overall, technology use was frequent. Of the young people that had access to the various technologies, use varied according to technology type, as illustrated in Figure 1. Most young people (387/611, 63.3%) reported using their smartphones several times an hour, and a high proportion used social media (540/584, 92.5%), instant messaging (509/574, 88.7%), and computers (397/540, 73.5%) at least once or several times a day, with hourly use being the most common.

**Table 2:** A comparison of access to different technologies among young people from the general population, primary services, and specialist services and use of technology for clinical care among clinicians (N=693).

Technologies		Young people from the general population (n=327), n (%)	Young people from primary services (n=236), n (%)	Young people from specialist services (n=54), n (%)	Clinicians (n=76), n (%)
**Smartphone**		321 (98.2)	236 (100)	54 (100)	61 (80)
	iPhone	233 (71.2)	145 (61.4)	29 (54)	—^a^
	Android	88 (26.9)	91 (38.5)	25 (46)	—
Social media		316 (96.6)	222 (94.1)	46 (85)	4 (5)
Instant messenger		313 (95.7)	215 (91.1)	46 (85)	7 (9)
Laptop		306 (93.6)	197 (83.5)	37 (69)	55 (72)
Video chat		286 (87.4)	185 (78.4)	44 (81)	69 (91)
Gaming console		153 (46.8)	152 (64.4)	40 (74)	1 (1)
Tablet		117 (35.8)	84 (35.6)	15 (28)	27 (36)
Wearables		92 (28.1)	43 (18.2)	9 (17)	3 (4)
Desktop		80 (24.5)	66 (28)	16 (30)	43 (57)
Landline		75 (22.9)	57 (24.1)	13 (24)	33 (43)
Virtual reality		18 (5.5)	9 (3.8)	5 (9)	1 (1)

^a^Data not available.

#### Technology Use by Clinicians

**Figure 2:**
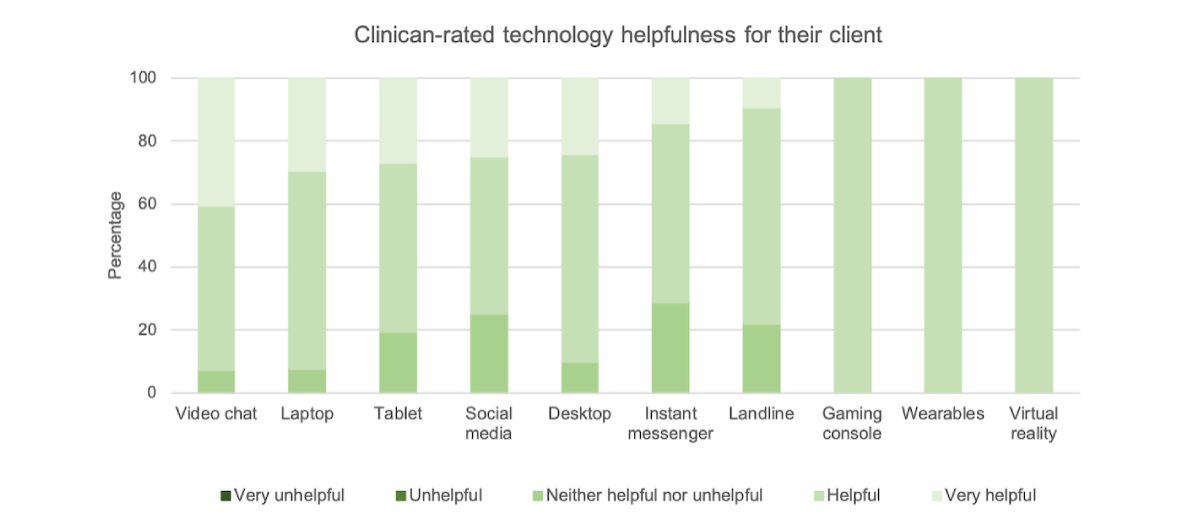
Clinicians’ perceived helpfulness of different technologies that they have used within clinical care (as presented in Table 2).

The proportion of clinicians who used different technologies in their clinical work is presented in Table 2. Although most clinicians used video chat (69/76, 91%) and smartphones (61/76, 80%) within their practice, few reported using newer or social technologies such as wearables (3/76, 4%), social media (4/76, 5%), or VR (1/76, 1%). The perceived helpfulness of the technology recommendations for clients is presented in Figure 2. Of the technologies used in clinical practice, most clinicians rated them helpful or very helpful for their clients.

#### Mental Health App Use

Approximately half of all participants (347/670, 51.8%) had used a mental health app themselves (young people: 296/609, 48.6%) or recommended one to their clients (clinicians: 51/61, 84%). A chi-square test for independence indicated a significant difference between participant groups and the use of apps for mental health (*χ*^2^_3_=28.8, n=670; *P*<.001; Cramer V=0.21), with clinicians significantly more likely to recommend apps to support mental health care than young people were to have used mental health apps. The percentage of young people and clinicians who had used apps for mental health and the most common apps used are presented in Table 3. These apps were similar across groups of young people and clinicians and were used for mindfulness, meditation and relaxation, mood monitoring, and safety planning.

**Table 3:** Young people’s use of smartphone apps and clinicians’ use or recommendations of smartphone apps for clients (N=670).

Participant groups	Used or recommended apps for mental health, n (%)	Most commonly used or recommended apps
Young people from the general population (n=319)	162 (50.8)	Smiling Mind, Headspace, Calm, and Calm harm
Young people from primary services (n=236)	111 (47)	Headspace, Smiling Mind, Calm, and Daylio
Young people from specialist services (n=54)	23 (43)	Calm, Headspace, Daylio, Smiling Mind, and YouTube
Clinicians (n=61)	51 (84)	Smiling Mind, BeyondNow, Headspace, and Calm

Overall, most young people from services (specialist services and primary care) reported that using apps to support their mental health was helpful or very helpful (82/132, 62.1%). Approximately 20.5% (27/132) neutral and 17.4% (23/132) reported apps to be unhelpful. Similarly, on average, young people from the general population found apps to be somewhat helpful (124/161, 77%). Approximately 13% (21/161) found them unhelpful. The vast majority of clinicians (45/48, 93.8%) felt that the apps were helpful to their clients.

#### Interest in Technology to Support Mental Health

**Figure 3:**
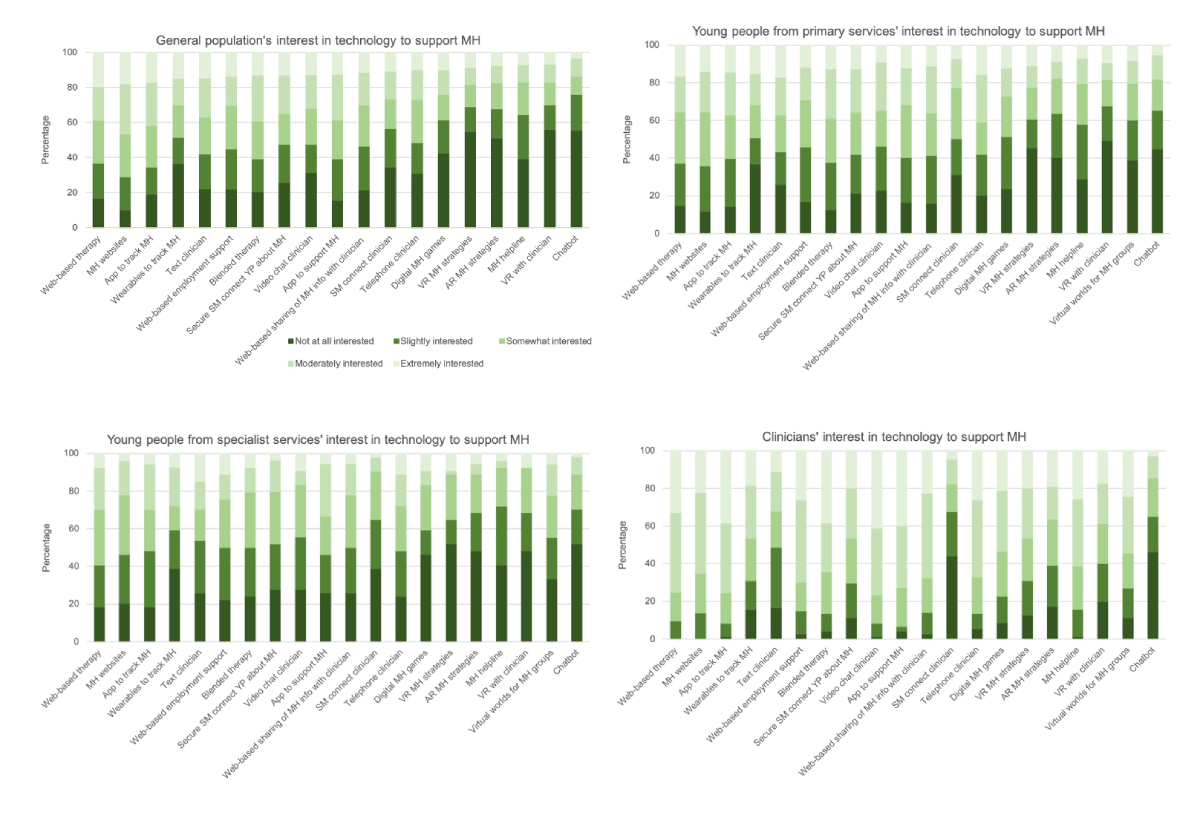
The average level of interest in different technological approaches to support mental health across the 4 participant groups: young people general population (n=306), young people primary services (n=229), young people specialist services (n=53), and clinicians (n=73). AR: augmented reality; MH: mental health; SM: social media; VR: virtual reality; YP: young people.

Young people’s and clinicians’ interest in different technologies to support mental health is presented in Figure 3. A Kruskal-Wallis test revealed a statistically significant difference in the overall level of interest in using technology to support mental health across the 4 participant groups (H_3_=55.90; *P*<.001). Follow-up tests with Bonferroni corrections were used to compare all pairs and indicated that clinicians were significantly more interested in using technology to support mental health than each of the groups of young people (general population: *χ*^2^_2_=−171.6, *P*<.001; primary services; *χ*^2^_2_=−158.7, *P*<.001; specialist services: *χ*^2^_2_=−218.9, *P*<.001). However, there was no significant difference in interest between young people in specialist services, primary services, or the general population (all *P*>.23). Although responses varied among the range of technologies surveyed, most participants (593/664, 89.3%) were at least slightly interested in a use of technology to support their mental health and well-being (young people: 520/591, 88%) or that of their clients (clinicians: 73/73, 100%). The technologies with the most consistently high levels of interest across these populations were telehealth, apps to track mental health, and web-based and blended therapies. The lowest level of interest overall was for chatbots, wearables, and immersive technologies (VR and augmented reality). However, close to half of all respondents across the sample reported at least some interest in all technology types.

**Figure 4:**
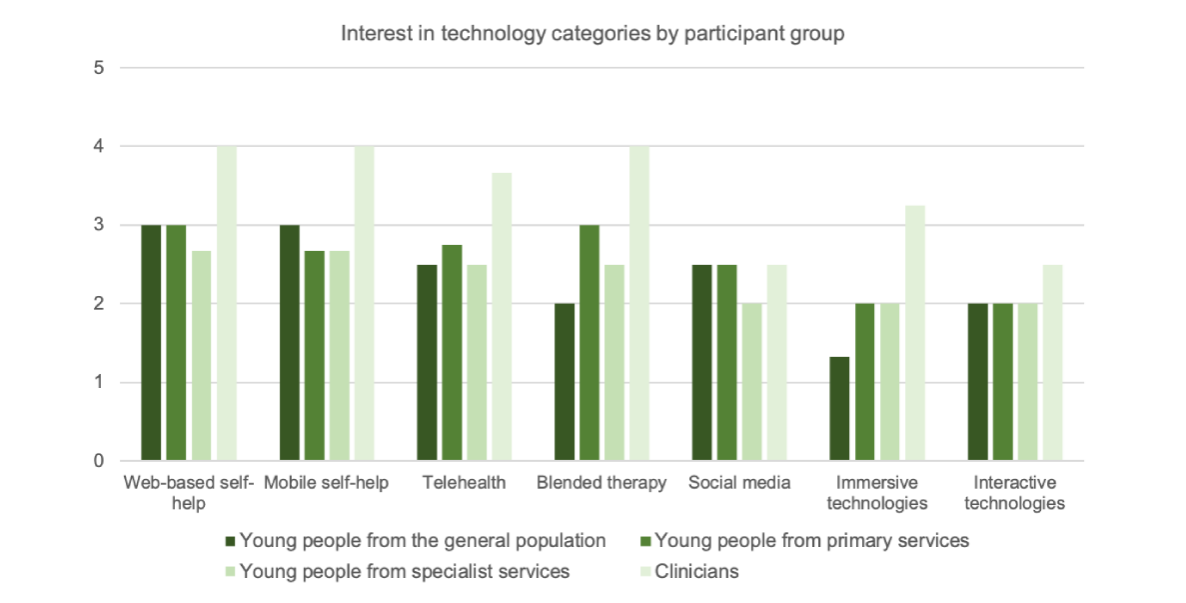
Level of interest in each of the participant groups for different categories of mental health technology.

Similar technology types were then grouped to observe the patterns of interest more clearly between the participant groups (Figure 4). Young people in the general population were most interested in web-based and mobile self-help, whereas young people from primary services were most interested in web-based self-help and blended therapy. Similar to the general population, those in specialist services were most interested in web-based and mobile self-help, as were clinicians, who also had high levels of interest in blended therapy.

Technology interest for mental health may be influenced by the respondents’ familiarity with the technology. A Mann-Whitney *U* test of independence found that young people who owned or had access to VR were significantly more interested in using VR to support their mental health than those who did not (*U*=5473.5; *z*=−2.811; *P*=.005).

## Discussion

### Principal Findings

This study examined the access and use of digital technologies among young people from youth mental health services and the general population, as well as the interest in digital technology use for supporting mental health care among young people and clinicians. The findings indicate that young people had widespread access to technologies, with 99% (611/617) having access to a smartphone and 63.3% (387/611) using it on average every hour. Clinicians reported similarly high rates of technology use to support their clinical care, with 91% (69/76) reporting the use of video chat, 80% (61/76) reporting the use of smartphones, and most finding common technologies such as laptops and the internet helpful or very helpful. Approximately 50% (296/609) of young people from within services and the general population reported using smartphone apps to support their mental health, and 84% (51/61) of the clinicians reported recommending them to their clients. Apps were reported to be helpful by 62.1% (82/132) of young people within services and 77% (124/161) in the general population. The vast majority of clinicians (45/48, 94%) found apps helpful for their clients. Levels of interest varied across different technologies for supporting youth mental health, although 100% (73/73) of clinicians were at least slightly interested in technology to support their clients’ mental health, and 88% (520/591) of the young people were interested in technology. There were particularly high rates of interest among young people in self-help tools such as smartphone apps, web-based therapies, and technologies integrated with routine care (*blended therapies* [2]). Young people from within clinical services and the general population did not differ in their interest in using technology to support mental health; however, clinicians had significantly higher levels of interest overall.

Rates of access to technology were high across young people from within and outside of youth mental health services, with 98% to 100% of those surveyed having access to an internet-enabled device such as a smartphone or computer. Furthermore, young people reported very frequent use of these technologies throughout their daily lives, averaging several times an hour for smartphones. This is in line with prior research showing access rates between 95% and 99% in youth populations within high-income countries [4,5], with young people describing they use these *almost constantly* [4]. Research into young people within youth mental health services has been limited, although some studies have found similar rates of approximately 90% within small clinical samples of young people with early psychosis [25,26]. The current findings add to this literature by demonstrating high rates of access and use of technologies within populations of young people who use youth mental health services, supporting the potential reach of DMHIs in this population.

Overall, 88% (520/591) of young people reported at least some interest in technologies to support their mental health and well-being, and this did not differ depending on whether they were using youth mental health services. However, the patterns of interest appeared to differ across groups. Although all young people showed high levels of interest in self-help technologies, particularly smartphone apps and web-based therapy, those from within the services were most interested in technologies that worked alongside a clinician, including blended therapies and telehealth. This highlights the perceived need among young people for technologies to support care delivery, a finding supported by research indicating that DMHIs are the most effective and engaging when used in conjunction with human support [20]. However, it is also important to note that access to youth mental health care is limited [34]; therefore, young people in the general population who may have an unmet need for care could rely more heavily on digital technologies as self-help tools to support their mental health. Young people within services, who, on the basis of the current findings, are likely to receive care that incorporates digital technology (ie, blended therapy), may have a greater appreciation for technology to support the care they are receiving. This highlights the differences in the needs of the 2 populations and the important role that both self-help and blended technologies play in meeting the demand for mental health support among young people. Furthermore, although levels of interest did not significantly differ overall, contextual factors such as the level and type of mental health support being sought (eg, low-intensity psychological treatment vs crisis intervention) or stage of care (eg, in remission vs acutely unwell) suggest that the needs and interests in different digital mental health technologies are likely to differ. For example, a relatively well young person who is in remission may be interested in smartphone-based symptom monitoring to prevent relapse, whereas a young person in active treatment may be interested in telehealth services and web-based therapy support.

Clinicians also endorsed high rates of interest in recommending a wide range of digital technologies to support youth mental health, with 100% (73/73) reporting at least some interest. Patterns of interest appeared to map well with young people, primarily for video calls, self-help apps, and web-based therapy. The most consistently endorsed technology across young people and clinicians was websites providing web-based therapy or mental health information and smartphone apps to track and support mental health. Indeed, 40% (29/73) of the clinicians were *extremely interested* in apps, and 33% (24/73) were extremely interested in web-based therapy, 38% (28/73) when used in a blended way. This aligns with most research and development that has occurred in digital mental health, particularly for smartphone apps [35], supporting the clear consumer demand for these products. Furthermore, research supports young people’s interest in blending technology with standard treatment as a way of increasing accessibility, continuity, and consolidation of treatment, as well as a means of accessing posttherapy support and strengthening the face-to-face relationship between clients and therapists [23]. However, there is a lack of evidence-based web-based therapies and smartphone apps currently available to support youth mental health [36], with some key exceptions [24,37], highlighting a critical area for further research and development.

In contrast, clinicians and young people were relatively less interested in automated therapies, such as chatbots, and technologies that made use of platforms that were infrequently accessed and used, such as VR. Although this may represent genuinely lower levels of interest in these technologies, it is also possible that this reflects a lack of familiarity and experience with their use for mental health treatment. Indeed, people tend to hold less positive attitudes and are less likely to adopt technologies with which they are less familiar [38]. This interpretation is supported by the finding that those who had used VR were significantly more interested in using it for mental health support. VR has a strong emerging evidence base for supporting the delivery of psychological interventions [39,40], particularly for exposure therapy; however, these interventions have not been widely implemented in clinical services. As the technology landscape is changing rapidly, levels of interest may increase as novel technologies such as VR become more common.

Clinicians also reported frequently using technology to support their practice, with 91% (69/76) using video chat, 80% (61/76) using smartphones, and >80% finding these helpful. Furthermore, overall, clinician interest in recommending digital technologies to support youth mental health was significantly higher than young people’s interest in using them (although both groups displayed high levels of interest). This finding is consistent with prior results from the BRACE survey, showing that 98% of youth mental health clinicians endorsed the ongoing use of telehealth beyond the COVID-19 pandemic [13]. However, these findings contrast with prior research findings that clinicians hold tentative views about the role of technology in mental health [41], particularly in regards to these replacing their care. Although a comparison sample is not available, the widespread adoption of technologies to support care delivery during the COVID-19 pandemic may account for the positive attitude change among clinicians. Therefore, the level of clinician interest is a positive finding, as the field seeks to promote the adoption of technologies within care systems, traditionally a challenge partly because of staff resistance [42,43]. The current findings may exemplify the *paradigm shift* in digital mental health arising from the global pandemic toward more digitally enhanced models of care [14,44]. This contemporary model of care has been heralded as potentially overcoming critical limitations of current mental health care systems; therefore, this shift brings about new hope for reform [45]. However, the degree to which this optimism will continue as the COVID-19 pandemic normalizes and the critical reliance on digital technology reduces is yet to be determined.

Half of the young people reported using smartphone apps for their mental health, and 84% (51/61) of the clinicians had recommended them to their clients, with most finding these helpful. This difference between young people and clinicians was statistically significant, indicating that although apps may be commonly recommended by clinicians, this does not correspond directly with uptake by young people. Given that young people have high levels of exposure to digital technologies within their everyday lives [4], it is likely that their motivation to use these for mental health arises from multiple sources, including social influences [46]. Indeed, research studies have found that both adults [47] and young people [48] with mental ill health most commonly use social media, searches (including Google and app store), and informal recommendations to select mental health apps. These prior studies also show that recommendations from friends and family were a more common source of mental health apps than recommendations from health care providers. Future research would benefit from exploring the best means of engaging and supporting young people in using evidence-based DMHIs for their mental health, particularly using participatory methodologies that involve young people as the ultimate end users of these products.

Notably, the apps most commonly used by clinicians and young people were those with significant market dominance. A recent app store review by Lau et al [35] found that 90% of mental health app downloads are accounted for by only 4 different apps (Headspace, Calm, Youper, and Wysa). Headspace and Calm were widely used in the current sample, as well as others supporting mindfulness or relaxation, mood tracking, and safety planning. With estimates that 325,000 health apps are currently available [35], the restricted range of apps being used highlights the driving force of marketing behind consumer choice and demand. However, strong marketing rarely translates to effectiveness, with only 2% of the available apps being supported by any sort of research evidence [35], and many have been found to undergo questionable ethical practices around privacy and security [49,50]. Furthermore, the apps most commonly used or recommended by clinicians and young people to support youth mental health were not specifically designed for this purpose. Given the importance of designing DMHIs for end users and ensuring they are backed by strong evidence, maximizing the benefits of technologies to help young people with mental health difficulties requires more research to develop, evaluate, and disseminate purpose-built solutions designed specifically for, and alongside, young people with lived experience of mental health difficulties [51]. In particular, there is a clear dearth of available smartphone apps designed to be integrated into clinical treatment, despite the clear interest in these products among young people using services and clinicians. As young people and clinicians have reported high levels of interest in blended therapies, it is surprising that there are very few digital technologies designed to support clinical care currently available, with some exceptions [37]. This highlights a critical discrepancy between what young people and clinicians want and what is available, which may reflect the challenges in implementing digital interventions in service settings. Informing efforts to implement evidence-based DMHIs to support clinical care is a critical area for future research [29,52].

### Strengths and Limitations

Although this study has a number of strengths, including its large sample of young people across the spectrum of need for care, the inclusion of clinicians as important additional stakeholders and end users of DMHIs, as well as the depth of the survey regarding different DMHIs, the findings should be interpreted with knowledge of study limitations. First, data were collected via technology; thus, respondents likely represent a sample of digitally enabled young people, and only a proportion of young people responded to the survey. A range of demographic factors such as income and education may have influenced young people’s access to, and beliefs about, technology; however, this information was not captured in this study. Importantly, particular populations of young people, such as those from culturally and linguistically diverse or low socioeconomic backgrounds, who may have a greater need for mental health care, may not be well represented in this survey because of lower rates of technology access in these populations. This was highlighted in another report from the BRACE survey as a primary consideration among clinicians regarding the suitability of DMHIs for some young people [13]. Other factors considered by clinicians included client willingness, access, and complexity of clinical presentation, highlighting the need for an individualized approach. This survey provides an overall picture of interest levels in DMHIs; however, there was clear variability within the sample. Understanding who these technologies are suited to, at what time, and in what context remains a critical area of future research to overcome the limitations of a *one size fits all approach*.

Second, we cannot guarantee that young people from the general population were not users of services or did not experience mental health issues. Indeed, the high rates of depression and anxiety reported in our general population sample indicate a potential need for care. However, we did not ask participants about their help seeking. Notably, these levels of mental health concerns match those of surveys conducted on the general Australian youth population during the pandemic, supporting the representativeness of the sample [53]. Third, there is an important distinction between clinicians’ recommendations for young people to use DMHIs and their use to support clinical care activities (ie, blended therapy). Additional research is required to gain insight into how clinicians use technology within the mental health treatment they provide and what technologies are most appealing to support their clinical work. Fourth, this survey was conducted at a time during which strict lockdown measures were instituted in Australia, limiting daily activities. Although technology use rates in this study were similar to populations of young people before the pandemic [4], it is possible that use rates increased in this sample during this time, as well as increased demand and interest in mental health support because of increased stress.

Finally, Australia is a high-income country with mental health services supported by government funding. Youth mental health services are free for young people, although capacity limitations and geographical barriers limit access to them. These results may not be generalizable to countries with more limited youth mental health services, in which the demand for and interest in DMHIs may be higher [54,55]. However, these findings establish a strong case in which young people across a spectrum of clinical needs are interested in DMHIs, and most have access to the technologies required to receive them.

### Conclusions

The global pandemic has brought forth a critical juncture in developing a new system of digitally enabled care that is aligned with the needs of those it intends to support. These findings provide valuable insights into the perspectives of clinicians and young people as end users of digital mental health technologies and provide a compelling case for further development and expansion of technologies to enhance youth mental health care.

## Appendix

Multimedia Appendix 1 Full survey on digital mental health use and interest among young people and clinicians.

## Abbreviations

DMHI: digital mental health intervention
GAD-2: General Anxiety Disorder-2
PHQ-2: Patient Health Questionnaire-2
VR: virtual reality
