# Supplementary material for: Ownership, Use of, and Interest in Digital Mental Health Technologies Among Clinicians and Young People Across a Spectrum of Clinical Care Needs: Cross-sectional Survey
Source: JMIR Ment Health. 2022 May 11;9(5):e30716. doi: 10.2196/30716 (PMC9133993; doi:10.2196/30716)
Supplement: Multimedia Appendix 1 [file mental_v9i5e30716_app1.docx]

**The impact of Covid-19 on youth mental health services - STAFF**

This survey forms an important part of our response to COVID-19. Staff are well aware that service delivery has changed dramatically as a result of COVID-19. It is important for us to understanding what impact this has had on young people and the quality of service we provide for them.

In this survey, we ask some questions about how changes due to COVID-19 have impacted service delivery and quality. Given the role that telehealth has played in the response to COVID-19, we are also interested in experiences with this technology and other ways that technology might be able to help.

The information you share will help us to make sure that we are doing our best to help young people who are a part of youth mental health services, including by informing policy, research and service improvement. Your information will be kept confidential. This survey will take about 10-15 minutes. We will aim to feedback the results of the survey soon after we finish collecting responses.

**Impact of COVID19 on service delivery and quality**

The following questions ask about how COVID-19 has impacted on the delivery and quality of services you provide to young people. Please answer as honestly as possible.

Where do you work? (single select)

1. Orygen Specialist services YAT
2. Orygen Specialist services Community
3. Orygen Specialist services other
4. Orygen primary services Access
5. Orygen primary services other

In the last week, please estimate how many young people have you tried to assess/review? (single select)

1. 0
2. 1-5
3. 6-10
4. 11-15
5. >15
6. Unknown

In the last week please estimate how many telephone reviews you have done? (single select)

1. 0
2. 1-5
3. 6-10
4. 11-15
5. >15
6. Unknown

In the last week please estimate how many video conferences you have done? (single select)

1. 0
2. 1-10
3. 11-20
4. 21-30
5. >30
6. Unknown

How many 'did not attends' did you have? (dropdown number)

What impact, if any, has COVID-19 had on the following aspects of the mental health care you provide within services? (Likert)

|  | **Very negative** | **Negative** | **Somewhat negative** | **None at all** | **Somewhat positive** | **Positive** | **Very positive** |
| --- | --- | --- | --- | --- | --- | --- | --- |
| 1. How well the care you provide meets the needs of your clients | _1_ | _2_ | _3_ | _4_ | _5_ | _6_ | _7_ |
| 1. How well the care you provide to your clients achieves what they want from it | _1_ | _2_ | _3_ | _4_ | _5_ | _6_ | _7_ |
| 1. How clinically effective the care is that you provide to your clients | _1_ | _2_ | _3_ | _4_ | _5_ | _6_ | _7_ |
| 1. How safe your client is with the care provided | _1_ | _2_ | _3_ | _4_ | _5_ | _6_ | _7_ |
| 1. How supported and respected your client is with the care provided | _1_ | _2_ | _3_ | _4_ | _5_ | _6_ | _7_ |
| 1. How well coordinated and continuous the care you provide is | _1_ | _2_ | _3_ | _4_ | _5_ | _6_ | _7_ |
| 1. Whether your clients receive the care they need at the right time and place | _1_ | _2_ | _3_ | _4_ | _5_ | _6_ | _7_ |
| 1. How accessible and relevant the care is that you provide for your clients | _1_ | _2_ | _3_ | _4_ | _5_ | _6_ | _7_ |
| 1. How efficient and well organised the care is that you provide for your clients | _1_ | _2_ | _3_ | _4_ | _5_ | _6_ | _7_ |

On telehealth did you feel able to assess risk adequately? (single select)

1. Very well
2. Well
3. Hard to tell
4. Poorly
5. Very poorly

On telehealth did you feel able to provide clinical therapeutic work adequately? (single select)

1. Very well
2. Well
3. Hard to tell
4. Poorly
5. Very poorly

Are there additional admin burdens to clinicians in setting up telehealth options? Please estimate additional time per clinician (single sleect)

1. No additional mins
2. 1 - 30 mins
3. 31 mins - 60 mins
4. 1-2hrs
5. >2 hours
6. unknown

Are there additional admin burdens to admin in setting up telehealth? Please estimate additional time per admin per person (single sleect)

1. No additional mins
2. 1 - 30 mins
3. 31 mins - 60 mins
4. 1-2hrs
5. >2 hours
6. unknown

Did you spend less time with young people on telehealth than you would have in person? (single sleect)

1. Yes
2. Maybe
3. No

Please estimate the average amount of time per telephone call (single sleect)

1. Not applicable
2. 1-15 mins
3. 16-30 mins
4. 31-45 mins
5. 46-60 mins
6. >60 mins
7. unknown

Please estimate the average amount of time per video call (single sleect)

1. Not applicable
2. 1-15 mins
3. 16-30 mins
4. 31-45 mins
5. 46-60 mins
6. >60 mins
7. unknown

How many young people do you feel had difficulties as a result of telehealth? (drop down number)

- If more 1 or more, what were these difficulties (open)

What impact, if any, have changes in service delivery due to COVID-19 affected your client’s willingness or motivation to engagement with the service? (single sleect)

1. Very negative
2. Negative
3. Somewhat negative
4. None at all
5. Somewhat positive
6. Positive
7. Very positive

What impact, if any, have the changes in service delivery due to COVID-19 affected your relationship with your client? (single sleect)

1. Very negative
2. Negative
3. Somewhat negative
4. None at all
5. Somewhat positive
6. Positive
7. Very positive

How supported to you feel by senior staff to see a young person in person if indicated clinically? (single sleect)

1. Very unsupported
2. Unsupported
3. Neither supported nor unsupported
4. Supported
5. Very supported

Once services return to usual practices post-COVID-19, how interested would you be to continue using telehealth to deliver mental health care to some of your clients? (single sleect)

1. Not at all interested
2. Slightly interested
3. Somewhat interested
4. Moderately interested
5. Extremely interested

In considering whether you would offer telehealth to your clients in the future, what are the top four considerations you would have in making this decision? (open text)

What are the top four factors that would make you decide against using telehealth in future with your clients? (open text)

What advantages, if any, have you experienced as a result of changes in remote service delivery due to COVID19? (open text)

What disadvantages, if any, have you experienced as a result of changes in remote service delivery due to COVID19? (open text)

**Use of technology for service delivery**

#### These questions ask about technologies that you may have used during the service you provide to young people since the start of COVID19.

#### Which of the following technologies have you used to support the mental health of your clients or the care you have provided since the start of COVID19 (Monday 23 March, 2020)?

- Smartphone (a mobile device that can run apps and connect to the internet)
  1. If yes, then:
     1. Have you ever recommended or used a smartphone apps for your client’s mental health (Y/N)
        1. If yes, then:
           1. What was the name of the app you recommended or used? You can list multiple (open text)
           2. How helpful do you think this was for your client overall? (1 – not at all helpful, 2 – somewhat unhelpful, 3 – neither helpful nor unhelpful, 4 – helpful, to 5 – very helpful, 8 – unsure)
  2. No
  3. Don’t know
- Mobile phone (a mobile device that can make calls and send texts, but can’t run apps or connect to the internet)
  1. If yes, then:
     1. How helpful do you think this was for your client overall? (1 – not at all helpful, 2 – somewhat unhelpful, 3 – neither helpful nor unhelpful, 4 – helpful, to 5 – very helpful, 8 – unsure)
  2. No
  3. Don’t know
- Internet (e.g. websites, online programs)
  1. If yes, then:
     1. How helpful do you think this was for your client overall? (1 – not at all helpful, 2 – somewhat unhelpful, 3 – neither helpful nor unhelpful, 4 – helpful, to 5 – very helpful, 8 – unsure)
  2. No
  3. Don’t know
- Tablet (e.g. iPad)
  1. If yes, then:
     1. How helpful do you think this was for your client overall? (1 – not at all helpful, 2 – somewhat unhelpful, 3 – neither helpful nor unhelpful, 4 – helpful, to 5 – very helpful, 8 – unsure)
  2. No
  3. Don’t know
- Landline phone
  1. If yes, then:
     1. How helpful do you think this was for your client overall? (1 – not at all helpful, 2 – somewhat unhelpful, 3 – neither helpful nor unhelpful, 4 – helpful, to 5 – very helpful, 8 – unsure)
     2. Don’t know
- Wearables (e.g FitBit, Apple Watch)
  1. If yes, then:
     1. How helpful do you think this was for your client overall? (1 – not at all helpful, 2 – somewhat unhelpful, 3 – neither helpful nor unhelpful, 4 – helpful, to 5 – very helpful, 8 – unsure)
  2. No
  3. Don’t know
- Virtual reality
  1. If yes, then:
     1. How helpful do you think this was for your client overall? (1 – not at all helpful, 2 – somewhat unhelpful, 3 – neither helpful nor unhelpful, 4 – helpful, to 5 – very helpful, 8 – unsure)
  2. No
  3. Don’t know
- Laptop computer
  1. If yes, then:
     1. How helpful do you think this was for your client overall? (1 – not at all helpful, 2 – somewhat unhelpful, 3 – neither helpful nor unhelpful, 4 – helpful, to 5 – very helpful, 8 – unsure)
  2. No
  3. Don’t know
- Desktop computer
  1. If yes, then:
     1. How helpful do you think this was for your client overall? (1 – not at all helpful, 2 – somewhat unhelpful, 3 – neither helpful nor unhelpful, 4 – helpful, to 5 – very helpful, 8 – unsure)
  2. No
  3. Don’t know
- Video chat (e.g. Skype, FaceTime, Zoom, Whatsapp)
  1. If yes, then:
     1. How helpful do you think this was for your client overall? (1 – not at all helpful, 2 – somewhat unhelpful, 3 – neither helpful nor unhelpful, 4 – helpful, to 5 – very helpful, 8 – unsure)
  2. No
  3. Don’t know
- Social media (e.g. Facebook, Instagram, Twitter)
  1. If yes, then:
     1. How helpful do you think this was for your client overall? (1 – not at all helpful, 2 – somewhat unhelpful, 3 – neither helpful nor unhelpful, 4 – helpful, to 5 – very helpful, 8 – unsure)
  2. No
  3. Don’t know
- Instant messenger (e.g. WhatsApp, Facebook Messenger, SnapChat)
  1. If yes, then:
     1. How helpful do you think this was for your client overall? (1 – not at all helpful, 2 – somewhat unhelpful, 3 – neither helpful nor unhelpful, 4 – helpful, to 5 – very helpful, 8 – unsure)
  2. No
  3. Don’t know

### **Interest in digital mental health**

Technology can be used in different ways to help people with their mental health and wellbeing. We are interested in understanding how interested you would be in using or recommending the following technologies to support the mental health of your clients within the service. These are the final questions for this survey.

What is your level of interest in using or recommending the following technologies to support the mental health and wellbeing of your clients within the service?

|  | **Not at all interested** | **Slightly interested** | **Somewhat interested** | **Moderately interested** | **Extremely interested** |
| --- | --- | --- | --- | --- | --- |
| 1. Using a smartphone app for support with mental health and wellbeing | _1_ | _2_ | _3_ | _4_ | _5_ |
| 1. Using a smartphone app to keep track of mental health and wellbeing | _1_ | _2_ | _3_ | _4_ | _5_ |
| 1. Using a wearable device (e.g. FitBit) to keep track of mental health and wellbeing | _1_ | _2_ | _3_ | _4_ | _5_ |
| 1. Sharing information online about mental health and wellbeing with a clinician | _1_ | _2_ | _3_ | _4_ | _5_ |
| 1. Using video chat to talk to a clinician about mental health and wellbeing | _1_ | _2_ | _3_ | _4_ | _5_ |
| 1. Using voice chat (i.e. telephone) to talk to a clinician about mental health and wellbeing | _1_ | _2_ | _3_ | _4_ | _5_ |
| 1. Using text chat to talk to a clinician about mental health and wellbeing | _1_ | _2_ | _3_ | _4_ | _5_ |
| 1. Using social media to connect and interact with a clinician about mental health and wellbeing | _1_ | _2_ | _3_ | _4_ | _5_ |
| 1. Using technologies such as smartphone apps and computer programs in combination with therapy sessions with a clinician | _1_ | _2_ | _3_ | _4_ | _5_ |
| 1. Using a secure social media platform developed for mental health to connect and interact with other young people about mental health and wellbeing | _1_ | _2_ | _3_ | _4_ | _5_ |
| 1. Using websites to learn about mental health and wellbeing | _1_ | _2_ | _3_ | _4_ | _5_ |
| 1. Using an online therapy program to learn skills and strategies for mental health and wellbeing | _1_ | _2_ | _3_ | _4_ | _5_ |
| 1. Calling a telephone helpline (e.g. Lifeline, Beyond Blue) to talk to someone about mental health and wellbeing | _1_ | _2_ | _3_ | _4_ | _5_ |
| 1. Using structured online programs to learn skills and strategies for gaining work or enrolling in study | _1_ | _2_ | _3_ | _4_ | _5_ |
| 1. Speaking to a chatbot therapist (a computer application that engages in conversation via text) about mental health and wellbeing | _1_ | _2_ | _3_ | _4_ | _5_ |
| 1. Using virtual reality (wearing a headset to transport you to a virtual world) to learn skills and strategies for mental health and wellbeing | _1_ | _2_ | _3_ | _4_ | _5_ |
| 1. Using virtual reality to connect and interact with a clinician about mental health and wellbeing | _1_ | _2_ | _3_ | _4_ | _5_ |
| 1. Using augmented reality (projecting digital objects into the real world) to learn skills and strategies for mental health and wellbeing | _1_ | _2_ | _3_ | _4_ | _5_ |
| 1. Attending a psychosical group if they were run through a private online virtual world platform (e.g. SecondLife or The Sims) | _1_ | _2_ | _3_ | _4_ | _5_ |
| 1. Digital games that help you learn skills and strategies for mental health and wellbeing | _1_ | _2_ | _3_ | _4_ | _5_ |

This is the end of the survey. Thankyou for your time!

**The impact of Covid-19 on youth mental health services – YOUNG PEOPLE**

In this survey, we will ask some questions about some of the ways that COVID-19 may have impacted on your life, the quality of the service you receive from headspace, and some of the ways that technologies might be able to help. This survey will take about 15 minutes and your answers will help us to make sure that we are doing our best to help young people who are a part of headspace services.

**Demographics and screening**

How old are you? (number drop down)

What is your gender? (single select)

1. Male
2. Female
3. Transgender
4. Non-binary
5. Prefer not to say
6. Other, specify: _______________________
7. Unsure at this stage

Are you of Aboriginal or Torres Strait Islander background? (single select)

1. No
2. Aboriginal
3. Torres Strait Islander
4. Aboriginal and Torres Strait Islander
5. Prefer not to say

Which of the following best describes your employment situation status? (multi select)

1. Full time student
   1. If yes:
      1. Please indicate number of hours engaged in study per week: ______
2. Part time student
   1. If yes:
      1. Please indicate number of hours engaged in study per week: ______
3. Full-time worker in paid employment
   1. If yes:
      1. Please indicate number of hours engaged in work per week: ______
4. Part-time worker in paid employment If yes:
   1. If yes:
      1. Please indicate number of hours engaged in work per week: ______
5. Unpaid worker as a parent or carer
   1. If yes:
      1. Please indicate number of hours engaged in unpaid work per week: ______
6. Currently unemployed
   1. If yes:
      1. Please indicate number of hours engaged in job seeking per week: ______

What is your living situation currently? (single select)

1. Living with parents, caregivers, or siblings
2. Living with friends
3. Living with romantic partner
4. Living in shared accommodation
5. Living alone
6. Homeless or couch surfing

**Impact from Covid-19**

What impact, if any, has COVID-19 had on your study and/or employment/carers role? (Likert)

1. Very negative
2. Negative
3. Somewhat negative
4. None at all
5. Somewhat positive
6. Positive
7. Very positive

What impact, if any, has COVID-19 has on your non-work life? (Likert)

1. Very negative
2. Negative
3. Somewhat negative
4. None at all
5. Somewhat positive
6. Positive
7. Very positive

What impact, if any, has COVID-19 had on your mental health? (Likert)

1. Very negative
2. Negative
3. Somewhat negative
4. None at all
5. Somewhat positive
6. Positive
7. Very positive

In your own words, what have been the main *negative* impacts of COVID-19 on your mental health and wellbeing, if any? (open response)

In your own words, what have been the main *positive* impacts of COVID-19 on your mental health and wellbeing, if any? (open response)

**Impact on services**

What impact, if any, has COVID-19 had on the following aspects of the mental health care you receive from mental health services? (Likert)

|  | **Very negative** | **Negative** | **Somewhat negative** | **None at all** | **Somewhat positive** | **Positive** | **Very positive** |
| --- | --- | --- | --- | --- | --- | --- | --- |
| 1. How well the care you receive meets your needs | _1_ | _2_ | _3_ | _4_ | _5_ | _6_ | _7_ |
| 1. How well the care achieved what you wanted from it | _1_ | _2_ | _3_ | _4_ | _5_ | _6_ | _7_ |
| 1. How safe you feel with the care provided | _1_ | _2_ | _3_ | _4_ | _5_ | _6_ | _7_ |
| 1. How supported and respected you feel when receiving care | _1_ | _2_ | _3_ | _4_ | _5_ | _6_ | _7_ |
| 1. How well coordinated and continuous your care is | _1_ | _2_ | _3_ | _4_ | _5_ | _6_ | _7_ |
| 1. Whether you receive the care you need at the right time and place | _1_ | _2_ | _3_ | _4_ | _5_ | _6_ | _7_ |
| 1. How accessible and relevant your care is when you need it | _1_ | _2_ | _3_ | _4_ | _5_ | _6_ | _7_ |
| 1. How efficient and well organised the care is | _1_ | _2_ | _3_ | _4_ | _5_ | _6_ | _7_ |

How many telephone consult sessions with your clinician have you had since COVID-19 started (Monday 23 March, 2020)? (drop down number)

How many video consult sessions with your clinician have you had since COVID-19 started (Monday 23 March, 2020)? (drop down number)

Have you experienced any technological difficulties during remote consult sessions with your clinician? (single select)

- - - 1. Yes
      2. No

- If yes, what difficulties did you have? (open)

What impact, if any, have changes in service delivery due to COVID-19 affected your willingness or motivation to engage with the service? (Likert)

1. Very negative
2. Negative
3. Somewhat negative
4. None at all
5. Somewhat positive
6. Positive
7. Very positive

What impact, if any, have the changes in service delivery due to COVID-19 affected your relationship with your clinician? (Likert)

1. Very negative
2. Negative
3. Somewhat negative
4. None at all
5. Somewhat positive
6. Positive
7. Very positive

What advantages, if any, have you experienced as a result of the changes in service delivery due to COVID-19? (open)

What disadvantages, if any, have you experienced as a result of the changes in service delivery due to COVID-19? (open)

**Technology ownership and access**

### This next section asks you about your use of different technologies.

#### Which of the following do you personally own or have private access to? (multi select)

- Smartphone (a mobile device that can run apps and connect to the internet)
  1. If yes, then:
     1. What type of smartphone to you have? (single select)
        1. Android
        2. iPhone
     2. How often do you use this technology? (single select)
        1. Several times an hour
        2. Every hour/once an hour
        3. Once to several times a day
        4. Once to several times a week
        5. Less than once a week
     3. Have you ever used a smartphone app for your mental health (Y/N)
        1. If yes, then:
           1. What was the name of the app you have used most often for your mental health? (open question)
           2. How helpful did you find the app? (1 – not at all helpful, to 5 – very helpful)
  2. No
  3. Don’t know
- Mobile phone (a mobile device that can make calls and send texts, but can’t run apps or connect to the internet)
  1. If yes, then:
     1. How often do you use this technology? (single select)
        1. Several times an hour
        2. Every hour/once an hour
        3. Once to several times a day
        4. Once to several times a week
        5. Less than once a week
  2. No
  3. Don’t know
- Internet
  1. If yes, then:
     1. How often do you use this technology? (single select)
        1. Several times an hour
        2. Every hour/once an hour
        3. Once to several times a day
        4. Once to several times a week
        5. Less than once a week
  2. No
  3. Don’t know
- Tablet
  1. If yes, then:
     1. How often do you use this technology? (single select)
        1. Several times an hour
        2. Every hour/once an hour
        3. Once to several times a day
        4. Once to several times a week
        5. Less than once a week
  2. No
  3. Don’t know
- Landline phone
  1. If yes, then:
     1. How often do you use this technology? (single select)
        1. Several times an hour
        2. Every hour/once an hour
        3. Once to several times a day
        4. Once to several times a week
        5. Less than once a week
  2. No
  3. Don’t know
- Wearables (e.g FitBit, Apple Watch)
  1. If yes, then:
     1. How often do you use this technology? (single select)
        1. Several times an hour
        2. Every hour/once an hour
        3. Once to several times a day
        4. Once to several times a week
        5. Less than once a week
  2. No
  3. Don’t know
- Virtual reality
  1. If yes, then:
     1. How often do you use this technology? (single select)
        1. Several times an hour
        2. Every hour/once an hour
        3. Once to several times a day
        4. Once to several times a week
        5. Less than once a week
  2. No
  3. Don’t know
- Laptop computer
  1. If yes, then:
     1. How often do you use this technology? (single select)
        1. Several times an hour
        2. Every hour/once an hour
        3. Once to several times a day
        4. Once to several times a week
        5. Less than once a week
  2. No
  3. Don’t know
- Desktop computer
  1. If yes, then:
     1. How often do you use this technology? (single select)
        1. Several times an hour
        2. Every hour/once an hour
        3. Once to several times a day
        4. Once to several times a week
        5. Less than once a week
  2. No
  3. Don’t know
- Video chat (e.g. Skype, FaceTime, Zoom, Whatsapp)
  1. If yes, then:
     1. How often do you use this technology? (single select)
        1. Several times an hour
        2. Every hour/once an hour
        3. Once to several times a day
        4. Once to several times a week
        5. Less than once a week
  2. No
  3. Don’t know
- Social media (e.g. Facebook, Instagram, Twitter)
  1. If yes, then:
     1. How often do you use this technology? (single select)
        1. Several times an hour
        2. Every hour/once an hour
        3. Once to several times a day
        4. Once to several times a week
        5. Less than once a week
  2. No
  3. Don’t know
- Instant messenger (e.g. WhatsApp, Facebook Messenger, SnapChat)
  1. If yes, then:
     1. How often do you use this technology? (single select)
        1. Several times an hour
        2. Every hour/once an hour
        3. Once to several times a day
        4. Once to several times a week
        5. Less than once a week
  2. No
  3. Don’t know
- Gaming console (e.g Xbox, Nintendo Switch, Comp... Yes Is Selected
  1. If yes, then:
     1. How often do you use this technology? (single select)
        1. Several times an hour
        2. Every hour/once an hour
        3. Once to several times a day
        4. Once to several times a week
        5. Less than once a week
  2. No
  3. Don’t know

### **Interest in digital mental health**

Technology can be used in different ways to help people with their mental health and wellbeing. We are interested in understanding how young people feel about these different technologies.

#### What is your level of interest in using the following technologies for your mental health and wellbeing?

|  | **Not at all interested** | **Slightly interested** | **Somewhat interested** | **Moderately interested** | **Extremely interested** |
| --- | --- | --- | --- | --- | --- |
| 1. Using a smartphone app for support with mental health and wellbeing | _1_ | _2_ | _3_ | _4_ | _5_ |
| 1. Using a smartphone app to keep track of mental health and wellbeing | _1_ | _2_ | _3_ | _4_ | _5_ |
| 1. Using a wearable device (e.g. FitBit) to keep track of mental health and wellbeing | _1_ | _2_ | _3_ | _4_ | _5_ |
| 1. Sharing information online about mental health and wellbeing with a clinician | _1_ | _2_ | _3_ | _4_ | _5_ |
| 1. Using video chat to talk to a clinician about mental health and wellbeing | _1_ | _2_ | _3_ | _4_ | _5_ |
| 1. Using voice chat (i.e. telephone) to talk to a clinician about mental health and wellbeing | _1_ | _2_ | _3_ | _4_ | _5_ |
| 1. Using text chat to talk to a clinician about mental health and wellbeing | _1_ | _2_ | _3_ | _4_ | _5_ |
| 1. Using social media to connect and interact with a clinician about mental health and wellbeing | _1_ | _2_ | _3_ | _4_ | _5_ |
| 1. Using technologies such as smartphone apps and computer programs in combination with therapy sessions with a clinician | _1_ | _2_ | _3_ | _4_ | _5_ |
| 1. Using a secure social media platform developed for mental health to connect and interact with other young people about mental health and wellbeing | _1_ | _2_ | _3_ | _4_ | _5_ |
| 1. Using websites to learn about mental health and wellbeing | _1_ | _2_ | _3_ | _4_ | _5_ |
| 1. Using an online therapy program to learn skills and strategies for mental health and wellbeing | _1_ | _2_ | _3_ | _4_ | _5_ |
| 1. Calling a telephone helpline (e.g. Lifeline, Beyond Blue) to talk to someone about mental health and wellbeing | _1_ | _2_ | _3_ | _4_ | _5_ |
| 1. Using structured online programs to learn skills and strategies for gaining work or enrolling in study | _1_ | _2_ | _3_ | _4_ | _5_ |
| 1. Speaking to a chatbot therapist (a computer application that engages in conversation via text) about mental health and wellbeing | _1_ | _2_ | _3_ | _4_ | _5_ |
| 1. Using virtual reality (wearing a headset to transport you to a virtual world) to learn skills and strategies for mental health and wellbeing | _1_ | _2_ | _3_ | _4_ | _5_ |
| 1. Using virtual reality to connect and interact with a clinician about mental health and wellbeing | _1_ | _2_ | _3_ | _4_ | _5_ |
| 1. Using augmented reality (projecting digital objects into the real world) to learn skills and strategies for mental health and wellbeing | _1_ | _2_ | _3_ | _4_ | _5_ |
| 1. Attending a psychosical group if they were run through a private online virtual world platform (e.g. SecondLife or The Sims) | _1_ | _2_ | _3_ | _4_ | _5_ |
| 1. Digital games that help you learn skills and strategies for mental health and wellbeing | _1_ | _2_ | _3_ | _4_ | _5_ |

**Mental health**

Finally, we would like to know how you’ve been feeling over the past couple of weeks.

Over the last 2 weeks, how often have you been bothered by the following problems? (PHQ-4; screen depression and anxiety)

|  |  | **Not at all** | **Several days** | **More than half the days** | **Nearly every day** |
| --- | --- | --- | --- | --- | --- |
|  | Feeling nervous, anxious or on edge | _0_ | _1_ | _2_ | _3_ |
|  | Not being able to stop or control worrying | _0_ | _1_ | _2_ | _3_ |
|  | Little interest or pleasure in doing things | _0_ | _1_ | _2_ | _3_ |
|  | Feeling down, depressed or hopeless | _0_ | _1_ | _2_ | _3_ |

The following statements describe how people sometimes feel. For each statement, please indicate how often you feel the way described by checking the appropriate box. Here is an example: How often do you feel happy? If you never felt happy, you would respond "never"; if you always feel happy, you would respond "always." (UCLA Loneliness Scale – Brief)

|  | Statement | Never | Rarely | Sometimes | Always |
| --- | --- | --- | --- | --- | --- |
|  | I lack companionship | 1 | 2 | 3 | 4 |
|  | There is no one I can turn to | 1 | 2 | 3 | 4 |
|  | I am an outgoing person | 1 | 2 | 3 | 4 |
|  | I feel left out | 1 | 2 | 3 | 4 |
|  | I feel isolated from others | 1 | 2 | 3 | 4 |
|  | I can find companionship when I want it | 1 | 2 | 3 | 4 |
|  | I am unhappy being so withdrawn | 1 | 2 | 3 | 4 |
|  | People are around me but not with me | 1 | 2 | 3 | 4 |
